# Creation of Hollow Calcite Single Crystals with CQDs: Synthesis, Characterization, and Fast and Efficient Decontamination of Cd(II)

**DOI:** 10.1038/s41598-018-36044-5

**Published:** 2018-12-04

**Authors:** Tianli Yang, Ren He, Guihua Nie, Wenlei Wang, Gui Zhang, Yunchu Hu, Lichao Wu

**Affiliations:** 1 College of Science, Central South University of Forestry and Technology, Changsha, 410004 China; 2 Key Laboratory for Digital Dongting Lake Basin of Hunan Province, Changsha, 410004 China; 3 Key Laboratory of Cultivation and Protection for Non-Wood Forest Trees, Ministry of Education, Changsha, 410004 China

## Abstract

In this work, carbon quantum dots were first prepared through one-pot hydrothermal route of the propyl aldehyde and sodium hydroxide via an aldol condensation reaction, and a novel solid-phase extraction adsorbent of hollow calcite single crystals was prepared via the precipitation of metal nitrates by the CO_2_ diffusion method in the presence of CQDs and further applied for excessive Cd(II) ions removal from water. The spectra and morphologies of the etched calcite were investigated by X-ray diffraction, Fourier transform infrared spectrometry, Scanning electron microscope, and Transmission electron microscopy. The results show that the CQDs etching technique successfully furnish a strategy for manufacturing interface defects onto the calcite crystal. Bath studies were done to evaluate the effects of the major parameters onto Cd(II) adsorption by the etched calcite, such as pH, contact time, and initial Cd(II) concentration. The Cd(II) adsorption onto the new adsorbent could reach a maximum adsorption amount of 66.68 mg/g at 120 min due to the abundant exterior adsorption sites on the adsorbent. The adsorption kinetics and adsorption isotherms of Cd(II) on the etched calcite were also investigated. The experimental datum showed that the adsorption kinetics and isotherms of Cd(II) on the etched calcite were well-fitted by the pseudo-second-order kinetic model and the Freundlich isotherm model respectively. The adsorption mechanisms could be primarily explained as the formation of Cd(OH)_2_ and Ca_x_Cd_1−x_CO_3_ solid solution on the adsorbent surface with the help of X-ray photoelectron spectroscopy.

## Introduction

Toxic heavy metal contamination in the aquatic environment has been of great concern^1^. Because of its potential accumulation, this non-biodegradable and highly toxic metal present in the ecosystem and in humans i.e. cadmium (CdII), possesses harmful effects on all living organisms^2,3^. Therefore, it is very important to find a green and effective way to remove cadmium to an acceptable level^4,5^. At present, the methods of removing heavy metal ions^6–10^ are: chemical precipitation^11,12^, coagulation-flocculation-sedimentation^13^, solvent extraction^14,15^, ion exchange^16^, membrane filtration^17,18^ and adsorption^19,20^. Among them, adsorption is commonly considered to be one of the most promising techniques due to its apparatus simplicity, high efficiency process, and low costs^8,21–25^. A variety of novel materials, e.g., mesoporous material, carbonaceous material, magnetic material, molecular or ion-imprinted polymers, have been widely used in solid phase adsorption of heavy metals. Amines modified natural fibers were prepared via the hydrothermal method by using ethylene diamine and hydrazine as modifiers and fibers obtained from natural populus tremula as raw materials^26,27^. In recent decades, more and more researchers have described the adsorption process of Cd(II) ion and other heavy metal ions from aqueous solutions into various adsorbents^28–32^. Co-polymer of dimethyl diallyl ammonium chloride and dialylamin (PDDACD) was used to modify the films derived from the waste of palm date fruits, which were valuable materials for the treatment of industrial wastewater^33,34^.

Carbonates were recognized as good materials to remove heavy metals from industrial wastewaters and to immobilize metals. Calcite is one kind of carbonate mineral distributed widely in nature^35,36^. Some studies proved that Cd(II) could enter into the lattice of calcite and form (Cd Ca)CO_3_ solid solution, thereby achieving the removal of cadmium. Pérez-Garridoet *et al*.^37^ have investigated the interaction between calcite (10ī4) surfaces and Cd-bearing aqueous solutions. The growth of epilayers of the Cd_x_Ca_1−x_CO_3_ solid solution with Cd-rich members happened on the original calcite (10ī4) surface. There are many literatures to demonstrate that calcite has a certain ability to adsorb the heavy metal ions^38–40^. In consequence, how to increase the adsorption capacity of calcite has become a hot topic for the chemical researchers. Increasing the specific surface area may be an effective measure to increase the adsorption ability of calcite for heavy metals adsorption^41^. At present, the etching technology can effectively form interface lattice defects, thereby greatly increasing the specific surface area to provide more active sites and improve the adsorption capacity of heavy metal ions^42^.

As a new class of nanocarbon materials, carbon quantum dots (CQDs) contain numerous oxygen-containing functional groups^43^. CQDs are non-toxic with little harm to the environment, commonly used in biological imaging, photoelectric device, sensing and other fields. Taking this into account, CQDs are in prospect to turn into an alternative material for the removal of a variety of organic and inorganic pollutants owing to these oxygen-containing functional groups^44,45^. CQDs can modify inorganic metal compounds to improve some surface properties. Rahmanianet *et al*.^46^ combined LDHs with CQDs to fabricate tailored functional composite-based LDHs, so as to enhance the adsorption capacity. Thus, CQDs would provide a potential strategy for etching technology to form interface lattice defects and increase the specific surface area^47^.

In this study, the hollow calcite single crystals were first prepared by an etching technique with CQDs. They were characterized by using FT-IR, XRD, SEM and HR-TEM. The adsorption properties of the hollow calcite single crystals on Cd(II) have been investigated under the different conditions of pH (the pH change for 2, 3, 4, 5, 6, 7, 8), the contact time (0, 5, 15, 30, 60, 90, 120, 150, 180, 210, 240, 270, 300 min), and the initial concentration of Cd(II) solution (1, 5, 7, 8, 10, 15, 20, 25 mg/L), respectively. In the adsorption experiment, the volume of Cd(II) solution was 25 mL, the dosage of adsorbent was 10 mg, and the initial pH was detected by pH meter. The determination of cadmium ion concentration was performed by an inductively coupled plasma mass spectrometer (ICP-MS). Several adsorption models were selected to study the mechanism of kinetic adsorption and the adsorption isotherms. FT-IR, XRD, XPS, and HR-TEM were characterized to the interaction of Cd(II) with the hollow calcite single crystals. The results of this paper would provide insights to the mechanisms of the novel calcite single crystals on Cd(II) adsorption and will provide a potential and effective material for treating heavy metal ions in environment.

## Results and Discussion

### XRD and FT-IR analysis

**Figure 1:**
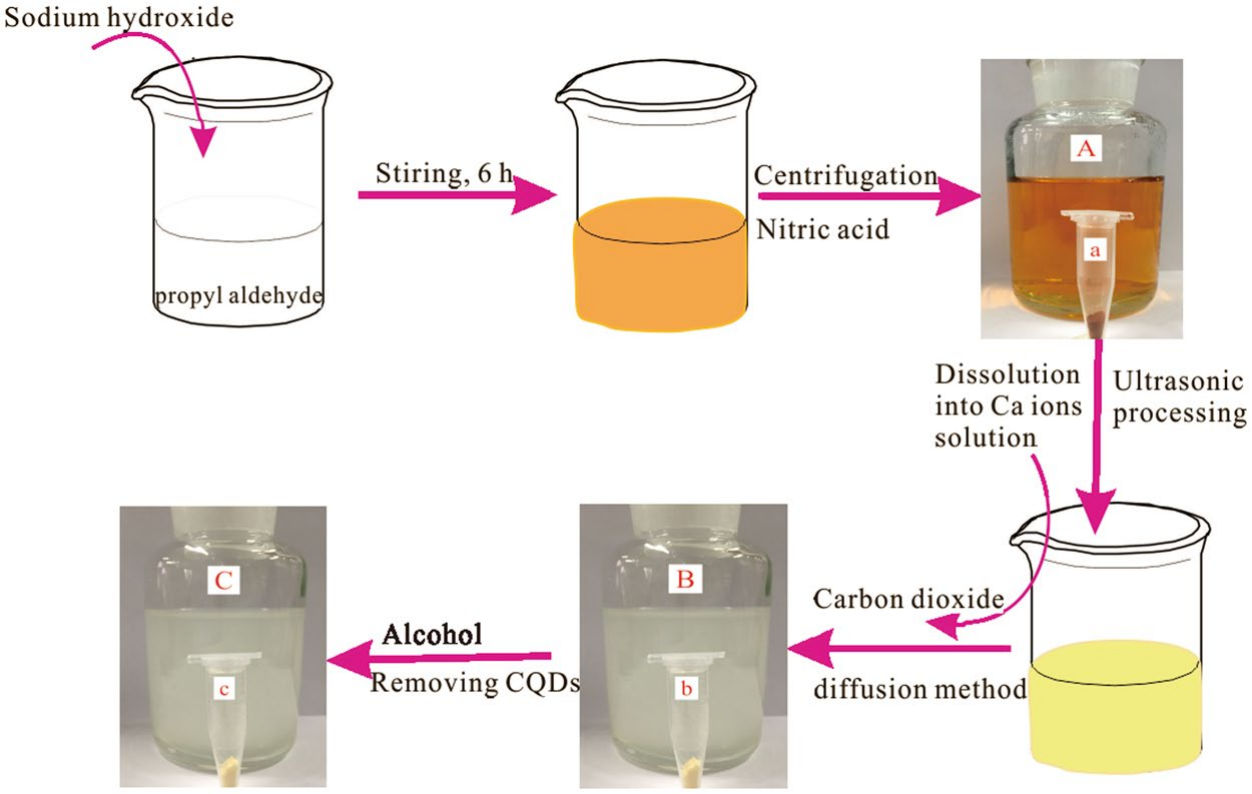
Synthesis of the etched calcite by a carbon dioxide diffusion method accompanying with etching technique. (**a**) CQDs; (**b**) CQDs/calcite; (**c**) the etched calcite.

**Figure 2:**
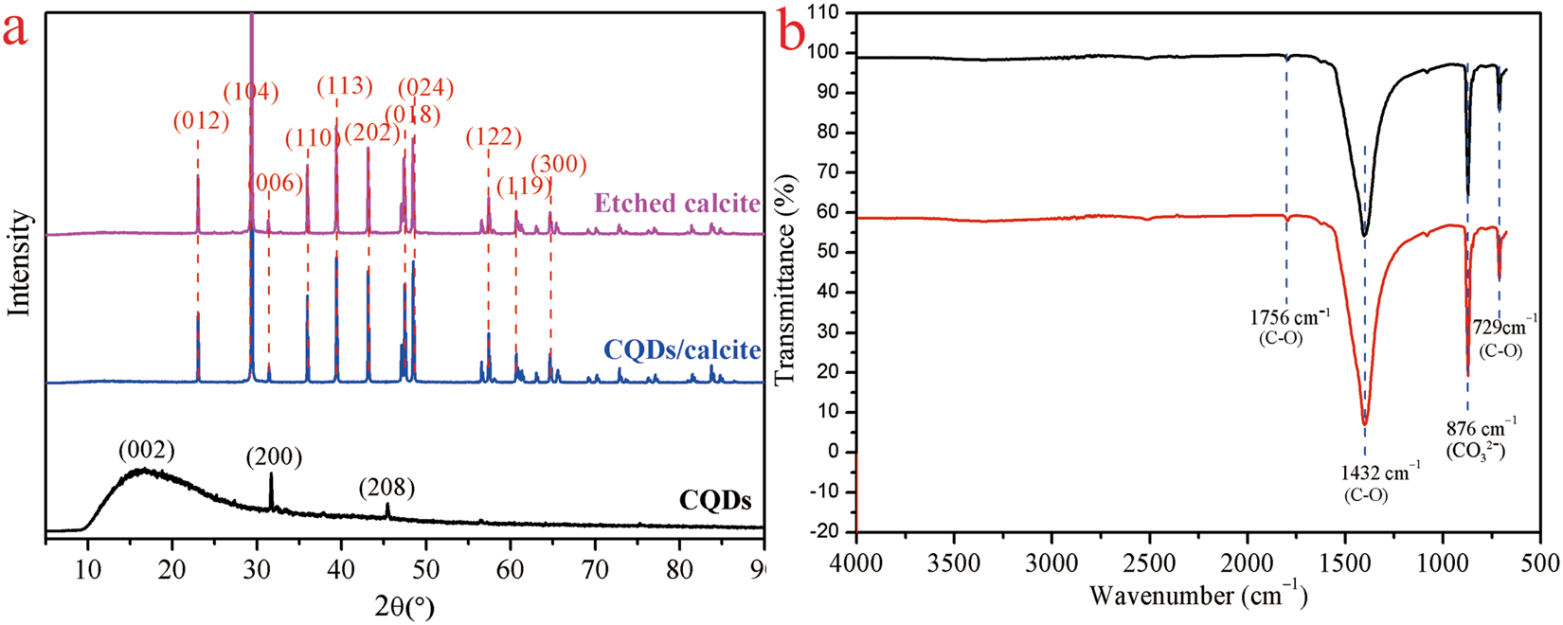
The XRD and FT-IR spectrums of the samples. (**a**) XRD patterns. black line: CQDs; blue line: CQDs/calcite; purple line: etched calcite. (**b**) FT-IR specta of the etched calcite before and after Cd(II) adsorption. black line: etched calcite; red line: etched calcite-Cd.

The preparation process of novel hollow calcite single crystal was shown in Fig. 1. The XRD patterns for the CQDs, the CQDs/calcite, and the etched calcite were presented in Fig. 2a. In the XRD pattern of the CQDs, a broad peak attributed to amorphous carbon, appeared near 2θ = 18°. In the XRD patterns of the CQDs/calcite and the etched calcite (as plotted in blue line and purple line in Fig. 2a), a typical rhombohedral phase of calcite was observed. It possessed a well-crystallized calcite structure with typical diffraction peaks related to (012), (104), (110), (113), (202), (018), (024), (122), (119), and (300) planes (conformed to JCPDS card 81–2027). (200) and (208) planes were the peak of NaCl (conformed to JCPDS card 75–0306). The reason for this might be that the CQDs would absorb a small amount of Na^+^ and Cl^−^ in the process of preparing CQDs. As can be seen, characteristic peak of (002) for the CQDs near 2θ = 18° had disappeared for the etched calcite. It can be illustrated that CQDs were almost completely erased from the CQDs/calcite. FT-IR spectra of etched calcite were shown in Fig. 2b. There were four adsorption bands, revealing the appearance of peaks at 729, 876, 1432, and 1756 cm^−1^ ^48^. The characteristic peaks at 729, 1432, and 1756 cm^−1^ for C-O stretching vibrations have been shown up in all spectrums. The adsorption bands appearing at 1432 cm^−1^ and 1756 cm^−1^ were associated with the C-O stretching vibration^49^ band and C-O antisymmetric stretching vibration, respectively. The peak at 876 cm^−1^ was associated for CO_3_^2−^ ^50^ contrasted with the FT-IR spectrums of the etched calcite before and after Cd(II) adsorption, where it was observed that the characteristic peaks didn’t change significantly.

### SEM analysis

**Figure 3:**
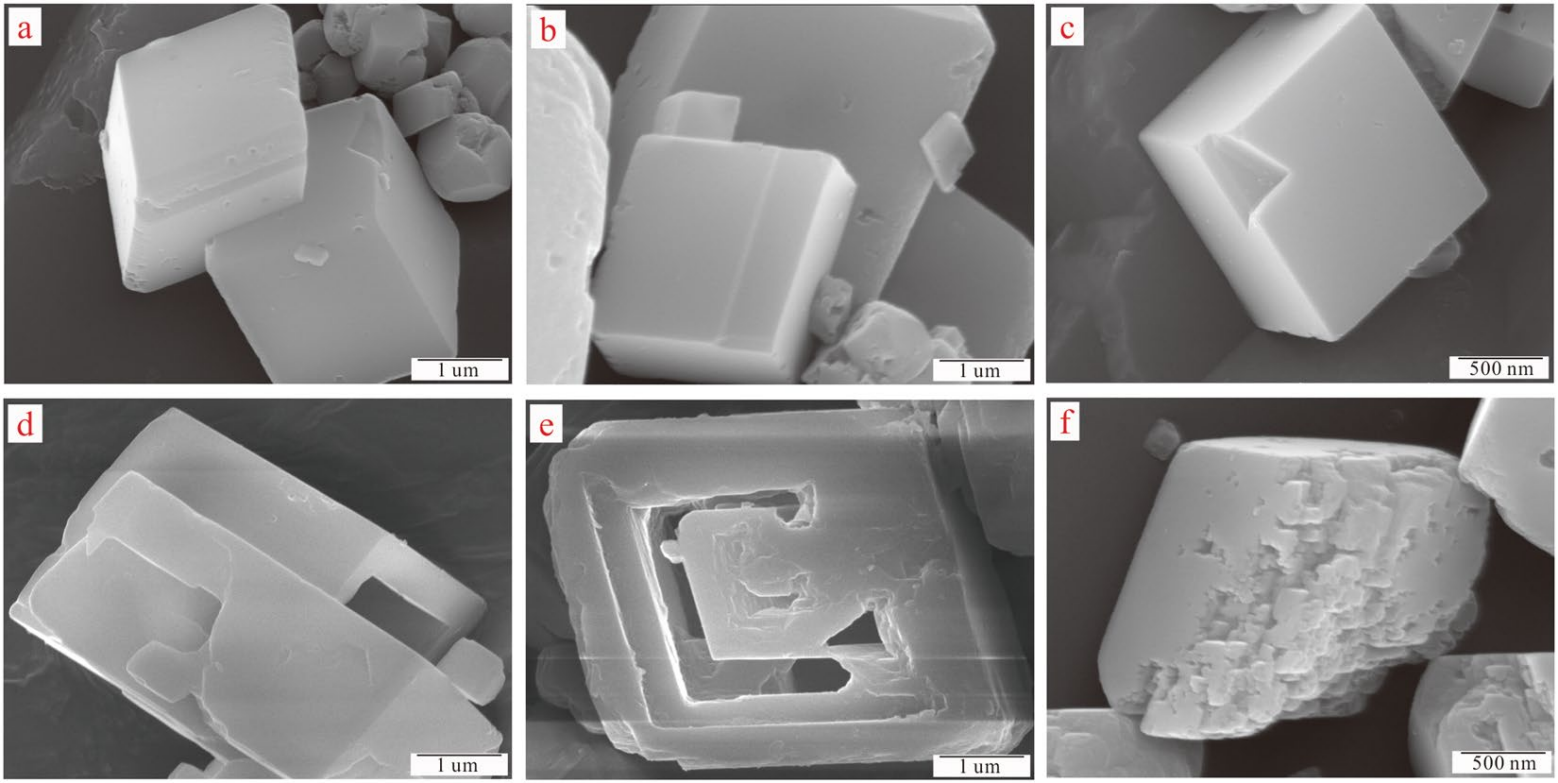
SEM images of the calcite before and after etching. (**a**–**c**) the calcite before etching; (**d**–**f**) the calcite after etching.

The SEM characterization was performed to study the morphologies of the calcite before and after etching, as shown in Fig. 3. SEM images of Fig. 3a–f presented the main crystal structural morphologies of the calcite and etched calcite, respectively. SEM images (Fig. 3a–c) represented three classical morphologies of the calcite. Other morphologies such as needle-like (aragonite) or spherulitic (amorphous calcium carbonate, ACC) have not been detected, which have a higher solubility than the crystalline phase^51^. As shown in Fig. 3a, it incarnated that the calcite before etching was interlaced growth and exhibited well developed rhombohedral crystals with sharp straight edges^39^. Figure 3b showed the complete and rhombohedral structure of calcite crystals that had been presented. As demonstrated in Fig. 3c, a certain surface defects had emerged on the surface of the calcite, while the defects were extremely inerratic. SEM images (Fig. 3d–f) presented three classical morphologies of the calcite after etching. The morphologies of the calcite after etching were obviously different from that of the calcite before. Figure 3d showed that the calcite after etching had interlaced growth with the rhombohedra crystal structure. Comparing this with Fig. 3a, a distinct hollow structure appeared in the interior of calcite after etching. By analysis, CQDs penetrated into the calcite in the synthesis process of CQDs/calcite and afterwards washed off by the ethanol solution. It could be observed from Fig. 3e that the calcite after etching formed relatively regular hollow in the interior of the calcite crystal. As shown in Fig. 3f, the calcite after etching had more and irregular surface defects. In conclusion, the interface defects of the etched calcite by an etching technique with CQDs may provide a possibility of increasing the specific surface area.

### TEM analysis

**Figure 4:**
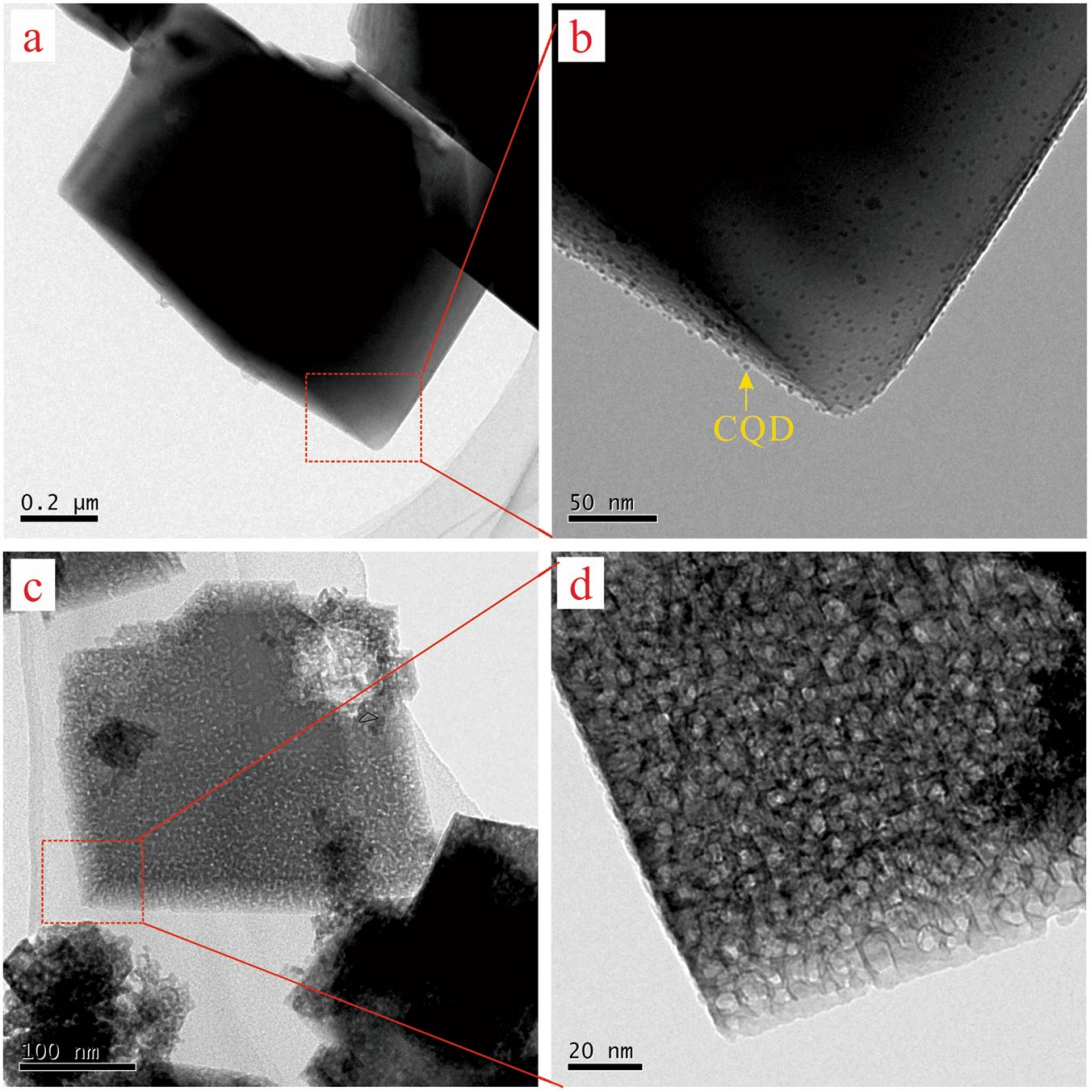
The TEM images of the calcite samples. (**a**,**b**) The CQDs/calcite; (**c**,**d**) the etched calcite.

TEM images (Fig. 4a,b) and others (Fig. 4c,d) presented the crystal structural morphologies of the CQDs/calcite and the calcite after etched, respectively. Figure 4a showed a rhombohedra morphology of the CQDs/calcite crystal. As illustrated in Fig. 4b, it is obvious that the CQDs were adhered and infiltrated into the interface of calcite crystal. Revealed in Fig. 4c,d, one would observe that the CQDs had been removed successfully from the generated calcite crystals, bringing about that the surface of the etched calcite very rough. A lot of etched holes have appeared on the surface and in the interior of the etched calcite crystal. These etched holes should be the original sites of the doped CQDs. Obviously, the interface defects of the etched calcite by an etching technique with CQDs may provide a possibility of increasing the specific surface area and supply more active sites for Cd(II) adsorption.

### Effect of pH

**Figure 5:**
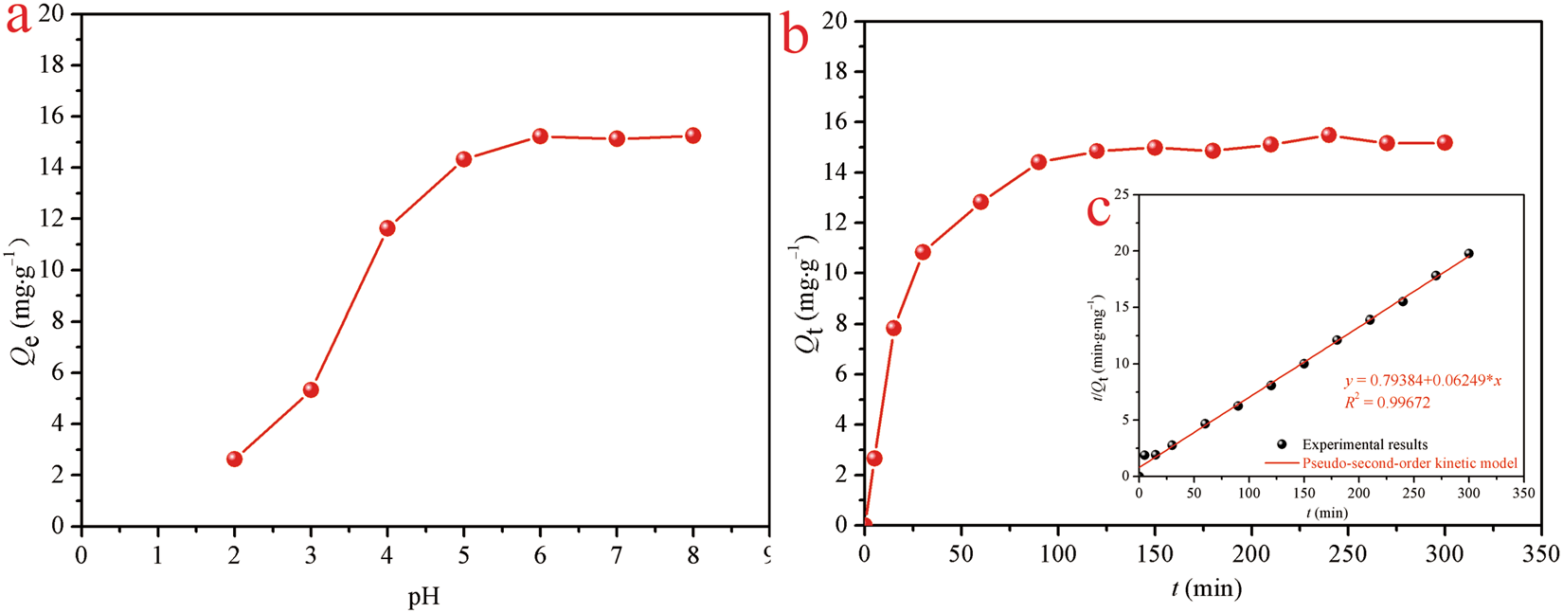
The effect of pH and contact time on Cd(II) adsorption onto the etched calcite. (**a**) pH (absorbent = 10 mg; *C*_Cd(II)_ = 10 mg/L; *V* = 25 mL; agitation speed = 180 rpm; contact time = 120 min). (**b**) Contact time (adsorbent = 10 mg; *C*_Cd(II)_ = 10 mg/L; *V* = 25 mL; agitation speed = 180 rpm; pH = 5). (**c**) Pseudo-Second-Order model.

The pH of the aqueous solution is an important criterion since it may affect the speciation of heavy metal ions through the formation of complexes or ligands, which, in turn influences the binding mechanism^52^. The solution pH plays a significant role in the Cd(II) adsorption process. For this purpose, the adsorption performance of Cd(II) on the etched calcite as a function of solution pH is often checked. In this study, the pH values above 9 were not studied due to the formation of the cadmium hydroxide precipitate (Cd(OH)_2_). With the etched calcite as adsorbent, the effect of pH on adsorption of Cd(II) ions was shown in Fig. 5a. As can be seen, it is found that the adsorption capacity of the adsorbent increased distinctly till a maximum value at pH 5.0, and then tended to be gentle above pH 5.0. pH could affect Cd(II) adsorption capacity in two ways, by influencing ion exchange and metal deposition reactions or by affecting the electric charge density of the surface to facilitate/hinder electrostatic interactions^53^. In terms of competition between H^+^ and Cd^2+^ and the protonation of the active sites of the adsorbent at a lower pH, the adsorption capacity was proved quite low at lower pH. The adsorption capacity raised up when pH arose as a result of weakening competition and waning repulsion^54^. Apparently, pH 5 was set as the optimized pH for subsequent adsorption experiments.

### Adsorption kinetics

To fully understand the dynamic interaction of metal cations with adsorbents and also predict the time required for adsorption equilibrium, the kinetics research of adsorption need to be carried out. The adsorption kinetics of Cd(II) on the etched calcite were studied with the results depicted in Fig. 5b. The adsorption capacity of the etched calcite raised up sharply in the first 60 min and followed by a relatively slower process to reach a maximum value after approximately 90 min. The plateau suggested that the adsorption has reached to equilibrium. The fast-initial uptake is frequently interpreted as being the result of chemisorption and the external surface adsorption and could be explained by that sufficient active sites on etched calcite. Whereas, the following slow removal is assumed to represent co-precipitation or surface precipitation^55–57^.

Conventional models have been tested for fitting experimental profiles transient studies^58,59^. The uptake kinetics have been modeled using the Pseudo-First Order rate equation (PFORE^59^) and the Pseudo-Second Order rate equation (PSORE^60^). Besides, in order to estimate the time-dependent intra-particle diffusion rate of Cd(II)from the surface sorption sites into the interior sites of etched calcite, Weber-Morris model^61^ was applied to define the adsorption kinetics mechanism.

The Pseudo-First-Order, Pseudo-Second-Order and Weber-Morris kinetic models are generally expressed as Eqs (1), (2), (3), respectively^62,63^:

1 $${{Q}}_{t}={{Q}}_{e}(1-{{e}}^{-{{K}}_{1}{t}})$$

2 $${{Q}}_{t}=\frac{{{K}}_{2}{{{Q}}_{e}}^{2}{t}}{1+{{K}}_{2}{{Q}}_{e}{t}}$$

3 $${{Q}}_{t}={{K}}_{{\rm{ip}}}{{t}}^{1/2}+{C}$$

Linearizing forms of the former two kinetic models can be demonstrated in Eqs (4) and (5), respectively.

4 $$\mathrm{ln}\,({{Q}}_{e}-{{Q}}_{t})=\,\mathrm{ln}\,{{Q}}_{e}-{{K}}_{1}{t}$$

5 $$t/{{Q}}_{t}=1/{{K}}_{2}{{{Q}}_{e}}^{2}+t/{{Q}}_{e}$$

where, *K*_1_ (min^−1^) is defined the rate constant of Pseudo-First-Order, and *K*_2_ (g/(mg·min)) is the rate constant of Pseudo-Second-Order models. *K*_id_ is defined the intra-particle diffusion rate constant (g/mg·min^0.5^). *C* is the intercept of Weber-Morris model.

The Pseudo-Second-Order kinetic model was used to describe the experimental results using Eq. (7). The calculated results of the above three models were presented in Fig. 5c and listed in Table 1, respectively. Based on the results, the Pseudo-Second-Order model presented the better correlation coefficient values than those of the Pseudo-First-Order model and Weber-Morris kinetics (*R*^2^: the Pseudo-Second-Order model (0.9967) > the Pseudo-First-Order model (0.9932) > the Weber-Morris model (0.7811)). Furthermore, the estimated *Q*_e_ values possess high self-consistency with the theoretical ones. It could be concluded that the dominant mechanism was the chemical adsorption^64^.

**Table 1:** Rate constants of the Pseudo-First-Order, Pseudo-Second-Order and Weber-Morris kinetics models for adsorption of Cd(II) onto the etched calcite.

Adsorbent	Pseudo-First-Order			Pseudo-Second-Order			Weber-Morris		
	*K*_1_ (min^−1^)	*Q*_e_(mg/g)	*R* ^2^	*K*_2_ (min^−1^)	*Q*_e_(mg/g)	*R* ^2^	*K*_p_	*C*	*R* ^2^
Cd(II)	0.0426	14.9850	0.9932	0.0035	16.5144	0.9967	0.8178	3.7321	0.7811

### Adsorption isotherms

**Figure 6:**
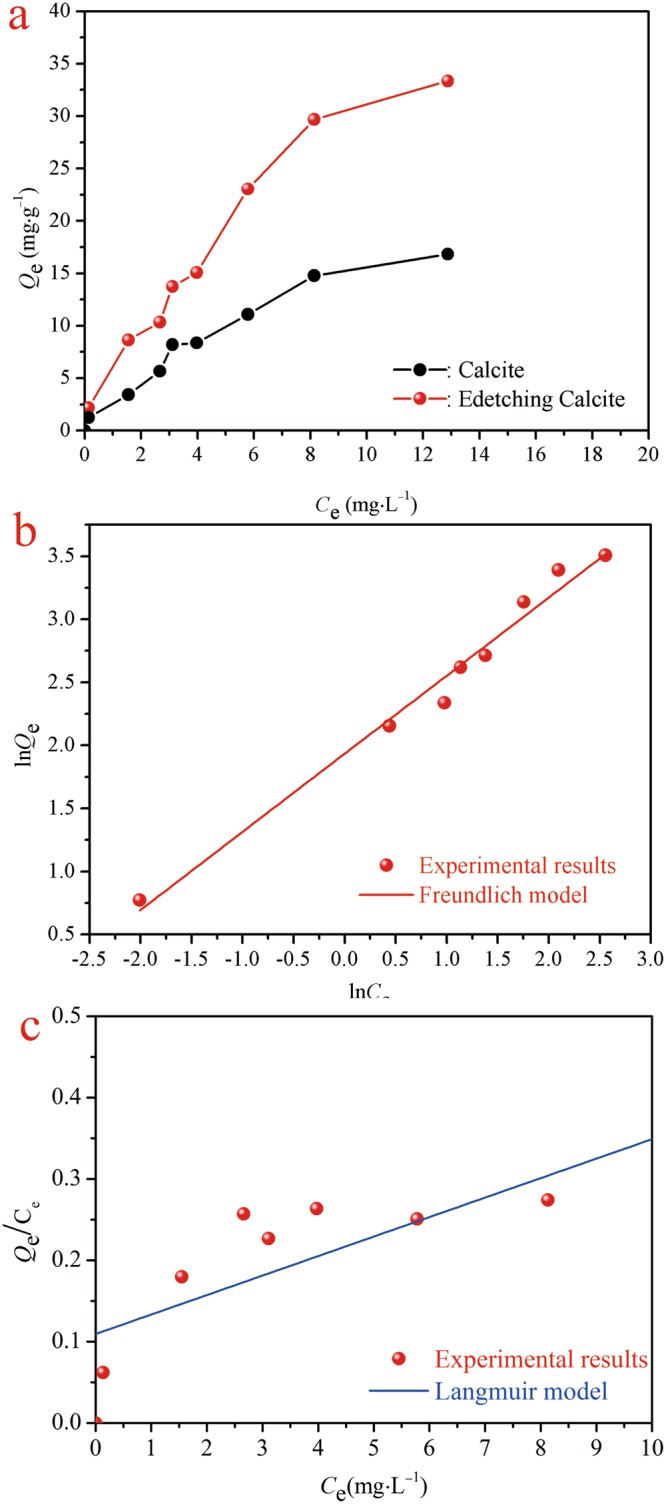
Adsorption isotherm of Cd(II) ions onto the etched calcite. (**a**) Adsorption isotherm (adsorbent = 10 mg; *V* = 25 mL; agitation speed = 180 rpm; pH = 5; contact time = 120 min). (**b**) Freundlich model; (**c**) Langmuir model.

Adsorption thermodynamics were investigated to evaluate the adsorption performance of the etched calcite towards Cd(II), through which the relationship between absorbent concentration and adsorption capacity was further studied. The adsorption isotherms were presented in Fig. 6a. The adsorption capacity of the etched calcite raised up with the increasing of the initial concentration of Cd(II). The amount of adsorption increased slowly with continuously increasing the initial Cd(II) concentration. In this study, the etched calcite for the adsorption capacity of Cd(II) adsorption could reach 29.68 mg/g with the initial concentration of 20 mg/L solution.

Most adsorption datum can be suitably expressed using either the Langmuir model^65^ or the Freundlich model^66^. The original equation and the linearized form of these models can be expressed in the Eqs (6), (7), (8) and (9), respectively^67–69^.

6 $${{Q}}_{{\rm{e}}}=\frac{{{Q}}_{{\rm{m}}}{{K}}_{{\rm{L}}}{{C}}_{{\rm{e}}}}{1+{{K}}_{{\rm{L}}}{{C}}_{{\rm{e}}}}$$

7 $${{Q}}_{{\rm{e}}}={{K}}_{{\rm{F}}}{{{C}}_{{\rm{e}}}}^{1/{n}}$$

8 $${{C}}_{{\rm{e}}}/{{Q}}_{{\rm{e}}}=1/({Q}{}_{{\rm{m}}}{K}_{{\rm{L}}})+{{C}}_{{\rm{e}}}/{{Q}}_{{\rm{m}}}$$

9 $$\mathrm{ln}\,{{Q}}_{{\rm{e}}}=\,\mathrm{ln}\,{{K}}_{{\rm{F}}}+(1/{n})\mathrm{ln}\,{{C}}_{{\rm{e}}}$$

where, *Q*_m_ is the maximum sorption capacity (mg/g), and *K*_L_ is defined a constant related to binding energy of the sorption system (L/mg). *K*_F_ ((mg/g)·(mg/L)^1/n^) is defined the Freundlich constant linked with the relative capacity. *n* corresponds with adsorption intensity^70^.

The thermodynamic models were used to describe the experimental results using Eqs 8 and 9 with the results plotted in Fig. 6b,c and the model parameters listed in Table 2. The Freundlich model better fitted the experimental results than Langmuir model. It indicated that the adsorption of Cd(II) onto the heterogeneous surface of the etched calcite belonged to the multilayer adsorption. The obtained amount of *n* was 1.5207 from the slope (1/*n*) 0.6576, indicating a favorable adsorption process (1 < *n* < 10)^71^.

**Table 2:** Parametersof Langmuir and Freundlich isotherm models for Cd(II) adsorption by the etched calcite.

Adsorbent	Langmuir constants			Freundlich constants		
	*Q*_max_(mg/g)	*K*_1_ (L/mg)	*R* ^2^	*K*_F_ (mg/g)(mg/L)^1/n^	1/*n*	*R* ^2^
Cd(II)	66.68	0.0845	0.9798	6.6223	0.6576	0.9792

### Adsorption mechanisms

**Figure 7:**
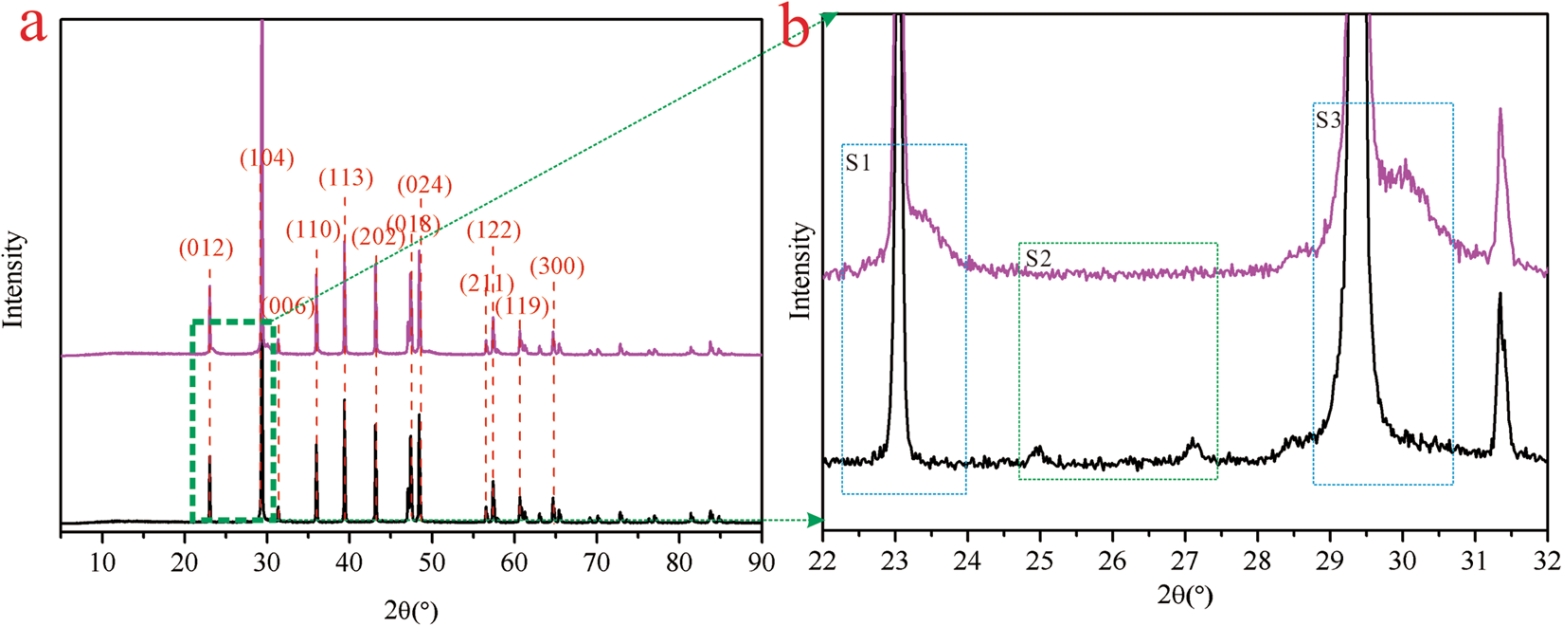
The XRD patterns of the etched calcite before and after Cd(II) adsorption. (**a**) Black line: etched calcite; purple line: etched calcite-Cd. (**b**) An enlarged view of the green area in (**a**).

The XRD patterns for the etched calcite before and after Cd(II) adsorption were presented in Fig. 7. As shown in Fig. 7a, the typical diffraction peaks of the etched calcite before and after Cd(II) adsorption possessed a well-crystallized structure related to the (012), (104), (110), (113), (202), (018), (024), (122), (119), and (300) planes (conformed to JCPDS card 81–2027). This might be because that both calcite and otavite are crystal structures of a trigonal system, and their lattice parameters are basically similar. However, there were some changes in the width or shape of typical diffraction peaks of the etched calcite before and after Cd(II) adsorption in three regions, which had been marked in Fig. 7b. In the regions of S1 and S3, the width of the typical diffraction perks (012) and (104) of the etched calcite after Cd(II) adsorption had dramatically increased compared with that of the etched calcite before Cd(II) adsorption. After adsorption, the Cd(II) ions might be penetrated into the interior of the etched calcite to form the (Ca, Cd)CO_3_ solid solution or rhombic cadmium^72^, consequently, the width of peaks of the etched calcite after adsorption of Cd(II) would be increased. In S2 region, some other diffraction peaks had disappeared on the interface of the etched calcite after Cd(II) adsorption, which was a calcite conformed to the JCPDS card 03–0612 with small content in the etched calcite.

**Figure 8:**
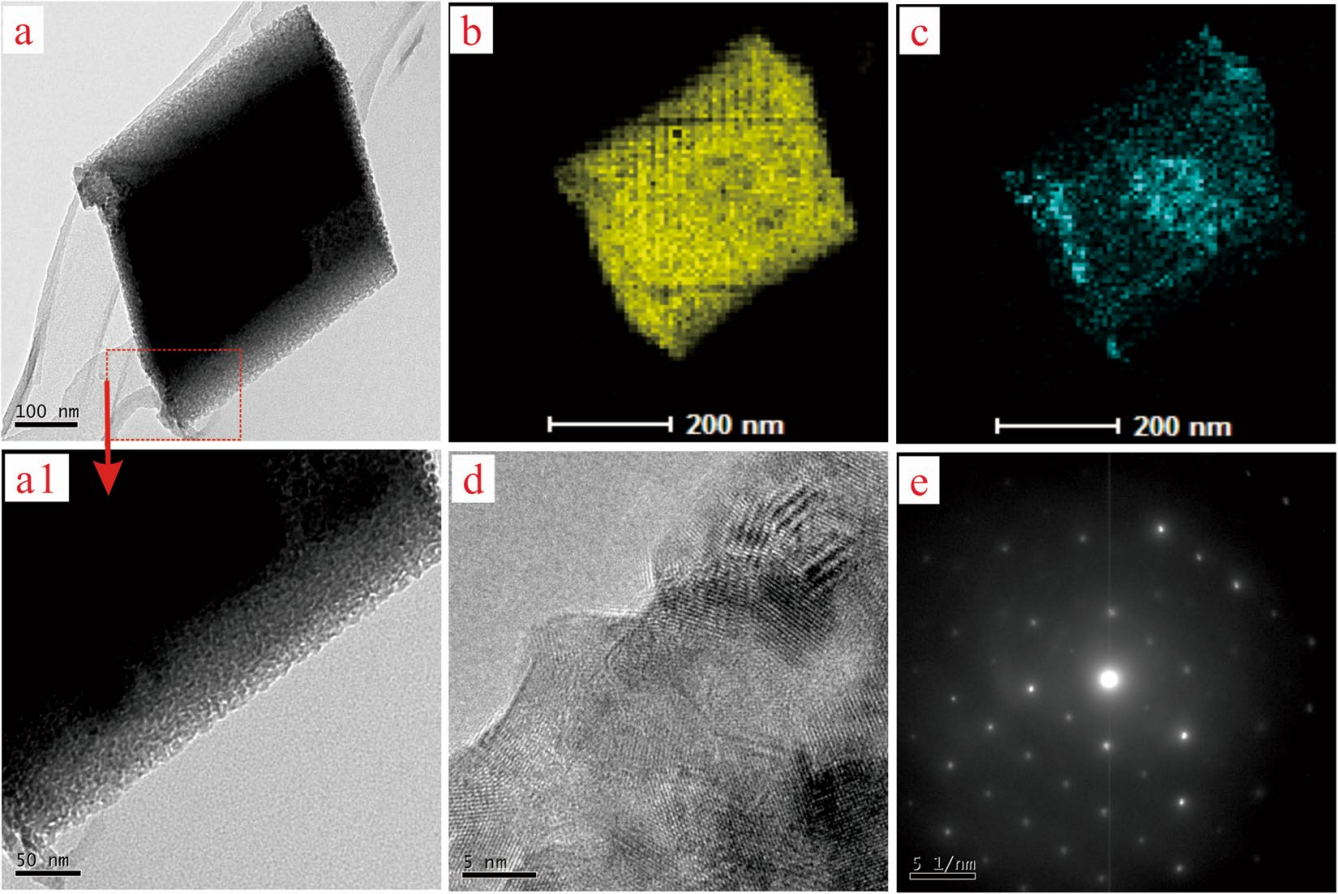
TEM images of the etched calcite after Cd(II) adsorption. (**a**) The TEM morphology; (**b**) the TEM-mapping image of Ca element; (**c**) the TEM-mapping image of Cd element; (**d**) HR-TEM image; (**e**) the morphology of diffraction pattern.

In order to study the adsorption behavior of Cd(II) onto the etched calcite, HR-TEM and TEM-mapping were applied to analyze the lattice distribution and element distribution of the etched calcite crystal after Cd(II) adsorption. HR-TEM and TEM-mapping images of the etched calcite in contact with the 10 mg/L solution of Cd(II) for 5 h were presented in Fig. 8. As displayed in Fig. 8a, the etched calcite after Cd(II) adsorption had crystal structure of rhombohedra and still existed in interface defects obtained by the etching technique. As can be observed in Fig. 8b,c, they represented the distribution of Ca element and Cd element in the etched calcite crystal after Cd(II) adsorption, respectively. The Cd element was evenly distributed on the surface of crystal. More cadmium element was distributed in certain areas of the etched calcite crystals interface, which illustrated that the area might be etched in higher degree. The TEM results proved that the adsorbed crystal might be the (Cd, Ca) CO_3_ solid solution. Callagon *et al*.^73^ observed that the (Cd, Ca) CO_3_ solid solution might emerge on the surface of calcite by AFM. The HR-TEM image and the pattern of single crystal diffraction were depicted in Fig. 8d,e, respectively. The lattices of the etched calcite after Cd (II) adsorption were distributed clearly, which meant that the Cd (II) did not result in the lattice defects of the etched calcite. And, diffraction patterns were distributed regularly. This was owing to that the ionic radius of Ca^2+^ (0.99 Å) is very close to that of Cd^2+^ (0.97 Å), and the electronic configurations of them formed in a similar way by losing the outermost electron from S atomic orbital^74,75^.

**Figure 9:**
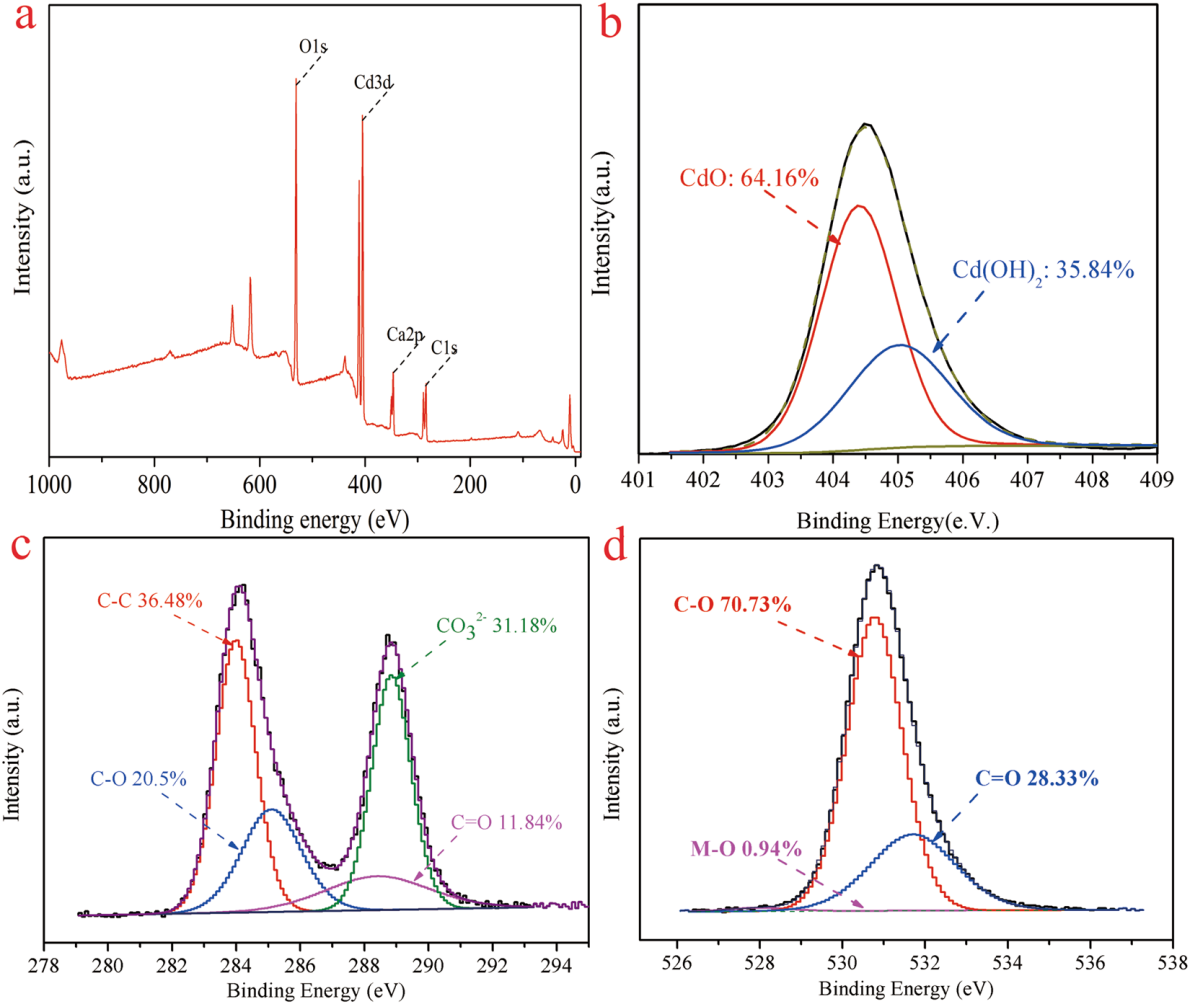
XPS spectra of the etched calcite after Cd(II) adsorption. (**a**) O 1 s, Cd 3d, Ca 2p, and C1s spectra of the etched calcite after Cd(II) adsorption; (**b**) Cd 3d core level spectra; (**c**) C 1 s core level spectra; (**d**) O 1s core level spectra.

The X-ray photoelectron spectroscopy (XPS) wide scan and different elements core-level spectra of the etched calcite after Cd(II) adsorption were employed to further investigate the function group information. The results of the XPS analysis were shown in Fig. 9. It revealed that the etched calcite after Cd(II) adsorption comprised a number of phases, including Cd(OH)_2_, CO_3_^2−^ and CdO. As observed in Fig. 9a, it shows that signal with binding energy of O 1 s Cd 3d, Ca 2p, and C were centered at 531 eV, 405 eV, 347 eV and 284 eV, respectively. this implies that the Cd(II) was successfully adsorbed by the etched calcite. As illustrated in Fig. 9b, two peak components of the Cd 3d core-level spectrum have binding energies at about 404.4 eV and 405 eV, which can be assigned to CdO (64.16 wt. %) and Cd(OH)_2_ (35.84 wt. %) species, respectively. The results clearly demonstrated that the deposition and chelation of Cd(II) played a significant role in the adsorption process^35^. The XPS C1s core-level spectrum with binding energy centered at about 284.043 eV, 285.154 eV, 288.446 eV, and 288.895 eV can be ascribed to C-C (36.48 wt. %), C-O (20.5 wt. %), C=O (11.84 wt. %), and CO_3_^2−^ (31.18 wt. %), respectively, (see Fig. 9c). As shown in Fig. 9d, three peak components of the O1s core-level spectrum have binding energies at about 530.692 eV, 531.516 eV, and 532.630 eV, which can be assigned to C=O (28.33 wt. %), C-O (70.73 wt. %), and M-O (0.94 wt. %), respectively. Combined Fig. 9b–d, it heralded that the existence of CdO may be in the form of cadmium carbonate (CaCO_3_) or cadmium calcium solid solution ((Cd, Ca)CO_3_).

## Conclusion

In this study, hollow calcite single crystals were synthesized by the etching technique with CQDs provide an extremely potential and important environmental protection material for removing cadmium ions from wastewater. By a series of pH experiments, it is found that the adsorption capacity of Cd(II) onto samples could reach the highest adsorption capacity at pH 5. In the kinetic adsorption study, the Cd(II) adsorption could reach adsorption equilibrium at 90 min. The process of Cd(II) adsorption onto the hollow calcite single crystals could be described as two steps. The fast initial uptake is frequently interpreted as being the result of chemisorption, whereas the following slow removal is assumed to represent surface precipitation or co-precipitation. In the thermodynamic adsorption study, the Cd(II) adsorption onto the hollow calcite single crystals could reach maximum adsorption amount at 66.68 mg/g. It manifested that the Cd(II) adsorption onto the etched calcite belonged to the chemical adsorption and multilayer adsorption in the form of CdCO_3_, Cd(OH)_2_, and (Ca, Cd)CO_3_ solid solution by the results of XRD, XPS and TEM. The novel hollow calcite single crystals will provide an efficient and environmentally friendly material for application into the removal of Cd(II) from wastewaters.

## Materials and Methods

### Materials

Nitric acid (AR, 65%), sodium hydroxide (AR, 90%), propyl aldehyde (AR, 99.5%), anhydrous ethanol (AR, 99.7%)and ammonium carbonate (AR, the NH_3_ content is not less than 40%) were purchased from Sinopharm Chemical Reagent Co., Ltd. Stock solutions of Ca(II) and Cd(II) of 1000 mg/L were prepared by dissolving a certain amount of calcium chloride anhydrous and cadmium nitrate tetra hydrate into distilled water and subsequently diluting to 1000 mL with deionized water (S < 1.5 × 10^−4^ S∙m^−1^), respectively, which were further confirmed by the inductively coupled plasma mass spectrometer (ICP-MS). The samples were weighed using a Sartorius BS224S balance with an error of ±0.1 mg. Deionized water (DIW) was used in all experiments.

### Synthesis of carbon quantum dots (CQDs)

In a typical procedure, CQDs were prepared as follows; as shown in Fig. 1. 2g of sodium hydroxide was slowly added into10 mL propyl aldehyde solution under the stirring condition at 20 °C for 6 h. The tawny colloidal sol would be obtained and then was laid in a sealed container for five days. After that, the tawny solid was rinsed with 15 mL of 0.5 M nitric acid to form yellow turbid liquid. Finally, the yellow turbid liquid was centrifuged for 15 min with a speed of 4000 rpm and dried at 60 °C for 6 h. The tawny solid prepared (as shown in Fig. 1a) was used for further experiments.

### Synthesis of etched calcite

The etched calcite was synthesized by a simple means, (see Fig. 1). In this typical synthesis, the synthetic steps were showed as follows: firstly, 0.4 g CQDs were added to 200 mL of 0.1 M Ca^2+^ aqueous solution and done ultrasonic treatment for 2 h with the purpose of making the CQDs completely dispersed into the Ca^2+^ aqueous solution; then, the mixed solution was placed in an airtight container with ammonium carbonate to release CO_2_ and supply CO_3_^2−^ for precipitate. In the process of CO_2_ diffusion, the mixed solution need be kept stirring constantly, so that CQDs and calcite could be combined completely; after 12 h, the CQDs/calcite solid sample obtained was centrifuged for 5 min with a speed of 4000 rpm and dried at 50 °C for 1 h. The flaxen solid sample of CQDs/calcite (as shown in Fig. 1b) was successfully prepared.

The characteristics of CQDs for being soluble in anhydrous ethanol would provide a feasible method for obtaining etched calcite. A certain amount of the flaxen solid sample obtained above was washed for four times with anhydrous ethanol solution. The off-white turbid liquid was then centrifuged for 5 min with a speed of 4000 rpm, and dried at 50 °C for 1 h to acquire the etched calcite. The off-white solid (as shown in Fig. 1c) prepared was used for the next adsorption experiments.

### Material characterization

A pH meter, with Amtast AMT12 (USA) model glass-electrode, was employed for measuring pH values of the aqueous phase. The chemical compositions were analyzed by an X-ray powder diffractometer (XRD) (Bruker D8 Advance, Germany) with Cu Kα radiation at 40 kV and 40 mA in a scanning range of 10°–90° (2θ). The surface functional groups were identified using a Fourier transform infrared spectrometry (FT-IR) spectrophotometer in range of 400–4000 cm^−1^ with the KBr disk method (Thermo Nicolet 5700, USA). Scanning electron microscope (SEM) images were recorded using a Philips-PEI model Quanta 200 with an accelerating voltage of 100 kV. High resolution transmission electron microscopy (HR-TEM) analysis was performed on a TecnaiG2 F20 S-TWIN for observing surface morphology and identifying of the elements of the samples. X-ray photoelectron spectroscopy (XPS) analysis were performed with an Axis Ultra spectrometer (Kratos Analytical Ltd.) using Al monochromatic X-ray source (Al Ka = 1486.6 eV) at 25 °C in a high vacuum environment (approximately 5 × 10^−9^ torr). All the binding energies were calibrated by using containment carbon the C1s (284.8 eV). The detection of cadmium ion was performed on an inductively coupled plasma mass spectrometer (ICP-MS, 5300DV, Perkin-Elmer, USA) by the standard addition method.

### Adsorption experiments

The adsorption experiments were performed in a thermostatic water bath oscillator (SHA-B, hannuo instruments) with a velocity of 180 rpm. A given 25 mL of Cd(II) aqueous solution at fixed concentration with an amount of 0.0100 g sorbent was used for the next adsorption experiments at 298.1 K.

The pH of the aqueous solutions has been identified as the most important parameter governing adsorption of metal ions on adsorbents. To determine the effect of pH parameter on Cd(II) adsorption with etched calcite as adsorbent, the pH from 2 to 8 of the aqueous solution were systematically carried out in the 10 mg/L of Cd(II) aqueous solution for the contract time of 120 min. The effect of contact time on Cd(II) adsorption from 0 to 300 min were performed in 10 mg/L Cd(II) solution. The effect of initial Cd(II) concentrations from 1 to 25 mg/L were investigated for the adsorption capacity. The pH values of the Cd(II) solutions were adjusted by adding 0.1 M HCl or 0.1 M NaOH until the desired pH was reached. After a period of time of adsorption, a suitable amount of solution was taken and diluted to the volumetric flask. A 0.45 µm syringe filter water membrane was applied to filter the suspension. The Cd(II) concentration was determined by ICP-MS.

In kinetic studies, the Cd(II) adsorption amount (*Q*_t_) could be determined by the following Eq. (10):

10 $${{Q}}_{t}=({{C}}_{0}-{{C}}_{t})\,{V}/m$$

where *C*_0_ and *C*_t_ (mg/L) refer to cadmium concentration at initial and *t* (min), respectively. *V* (L) is volume of Cd(II) solution. *m* (g) is Cd(II) adsorbent mass.

In thermodynamics studies, the adsorption capacity for cadmium uptake at equilibrium, *Q*_e_ (mg/g), could be calculated by the following Eq. (11):

11 $${{Q}}_{e}=({{C}}_{0}-{{C}}_{e}){V}/m$$

where *C*_e_ (mg/L) refer to Cd(II) concentration at equilibrium.
